# Paratransgenesis to control malaria vectors: a semi-field pilot study

**DOI:** 10.1186/s13071-016-1427-3

**Published:** 2016-03-10

**Authors:** Maria Vittoria Mancini, Roberta Spaccapelo, Claudia Damiani, Anastasia Accoti, Mario Tallarita, Elisabetta Petraglia, Paolo Rossi, Alessia Cappelli, Aida Capone, Giulia Peruzzi, Matteo Valzano, Matteo Picciolini, Abdoulaye Diabaté, Luca Facchinelli, Irene Ricci, Guido Favia

**Affiliations:** Scuola di Bioscienze e Medicina Veterinaria, Università di Camerino, Camerino, Italy; Department of Experimental Medicine, Centro di Genomica Funzionale, University of Perugia, Perugia, Italy; Institut de Recherche en Sciences de la Sante (IRSS), Direction Regionale de l’Ouest (DRO), BP 390 Bobo Dioulasso, Burkina Faso

**Keywords:** *Asaia*, *Anopheles*, Paratransgenesis, Large cages trials

## Abstract

**Background:**

Malaria still remains a serious health burden in developing countries, causing more than 1 million deaths annually. Given the lack of an effective vaccine against its major etiological agent, *Plasmodium falciparum*, and the growing resistance of this parasite to the currently available drugs repertoire and of *Anopheles* mosquitoes to insecticides, the development of innovative control measures is an imperative to reduce malaria transmission. Paratransgenesis, the modification of symbiotic organisms to deliver anti-pathogen effector molecules, represents a novel strategy against *Plasmodium* development in mosquito vectors, showing the potential to reduce parasite development. However, the field application of laboratory-based evidence of paratransgenesis imposes the use of more realistic confined semi-field environments.

**Methods:**

Large cages were used to evaluate the ability of bacteria of the genus *Asaia* expressing green fluorescent protein (*Asaia*^gfp^), to diffuse in *Anopheles stephensi* and *Anopheles gambiae* target mosquito populations. *Asaia*^gfp^ was introduced in large cages through the release of paratransgenic males or by sugar feeding stations. Recombinant bacteria transmission was directly detected by fluorescent microscopy, and further assessed by molecular analysis.

**Results:**

Here we show the first known trial in semi-field condition on paratransgenic anophelines. Modified bacteria were able to spread at high rate in different populations of *An. stephensi* and *An. gambiae*, dominant malaria vectors, exploring horizontal ways and successfully colonising mosquito midguts. Moreover, in *An. gambiae*, vertical and trans-stadial diffusion mechanisms were demonstrated.

**Conclusions:**

Our results demonstrate the considerable ability of modified *Asaia* to colonise different populations of malaria vectors, including pecies where its association is not primary, in large environments. The data support the potential to employ transgenic *Asaia* as a tool for malaria control, disclosing promising perspective for its field application with suitable effector molecules.

**Electronic supplementary material:**

The online version of this article (doi:10.1186/s13071-016-1427-3) contains supplementary material, which is available to authorized users.

## Background

The emergence of drug resistant parasites and insecticide resistant mosquito strains, together with several eco-environmental concerns related to the use of most chemicals, require the development of additional control methods for mosquito-borne diseases [1]. In addition to transgenic mosquitoes engineered to replace or suppress wild vector populations [2–4], a parallel approach aimed at producing paratransgenic tools to control vector-borne diseases has been developed, providing concrete possibilities for innovative control strategies [5–7].

Paratransgenesis is commonly defined as the use of symbiotic organisms, naturally inhabiting mosquito midgut and rapidly spreading among vector population, to deliver anti-pathogen effector molecules [8–10]. In the last decade several studies focusing on effective paratransgenic-based malaria control protocols have been published and a few bacterial symbionts have been already selected as potentially useful tools, although all related studies have been performed in small laboratory cages [11–15]. The transition from small laboratory cages to open field trials is a critical step to effectively set-up an in-depth control approach [16]. In this context, the intermediate step of confined semi-field conditions represents an ideal tool to evaluate the potential of paratransgenesis technology to be employed to counteract malaria and other mosquito-borne diseases. At the same time, it gives the possibility to develop predictive models and comprehensive risk assessment related to the use of paratransgenic mosquitoes. The use of large cages allows a wider picture of the actual transmission potential of selected symbiont(s), together with preliminary behavioural ecology insights of paratransgenic mosquitoes, in a specifically arranged environment, simulating the near-natural ecosystem conditions [17]. To our knowledge, no complete surveys in large cages have been yet performed for paratransgenesis.

The acetic acid bacterium *Asaia* is one of the most promising mosquito symbionts for paratransgenic approach. *Asaia* investigations in *Anopheles stephensi*, where it represents the dominant commensal genus, disclosed its ability to spread with high efficiency in recipient populations and throughout following generations, as demonstrated in small laboratory cages [18]. Moreover, the association between *Asaia* and field collected *An. gambiae* was reported [19]. Its intrinsic biological characteristics, easy transformability and capability to be transmitted through horizontal and vertical transmission routes in small cages, together with its colonisation throughout the mosquito life-cycle, as well as its co-localisation in *Plasmodium* invasion hot-spots, are invaluable features that make this bacterium a suitable candidate for symbiotic control strategies [20, 21].

**Fig. 1:**
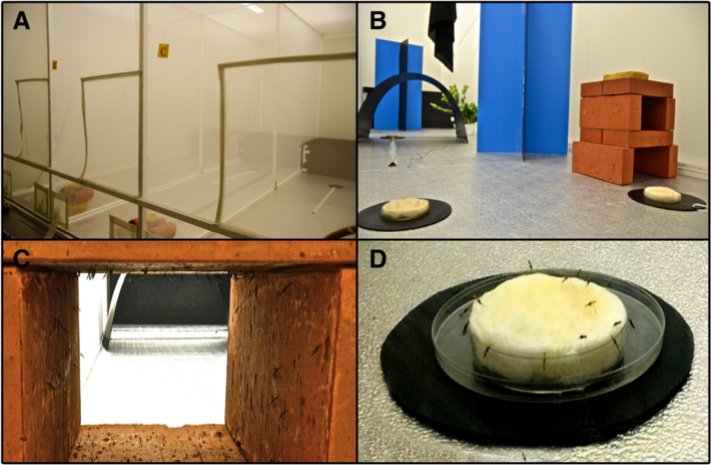
Illustration of the large cages in which experimental colonies were established. **a** View of the three cages. **b** Overall cage equipment including resting sites and swarming arena. **c** Mosquitoes resting in a clay brick and **d** sugar feeding station

Here we report the first known confined semi-field pilot trial with paratransgenic anopheline mosquitoes, carrying an *Asaia* expressing the Green Fluorescent Protein (*Asaia*^gfp^) [13], aimed to investigate the potential of this bacterium in paratransgenic approaches to control malaria and other mosquito borne diseases. The large cages present at University of Perugia (Italy), specifically equipped with clay brick resting sites, visual stimuli and mating areas for male swarming has already been proved to be suitable to perform behavioural and fitness studies of transgenic *An. gambiae* mosquitoes (Fig. 1) [22].

## Methods

### Mosquito rearing

Laboratory-strain colonies of *Anopheles gambiae* (G3) and *Anopheles stephensi* (SD500) were used. Mosquitoes were reared at 27 °C at a relative humidity of 70 %. Larvae were reared in deionised water to which 0.3 g/liter of artificial sea salts were added, and were fed daily with a diet provided as a slurry of 2:2:1 bovine liver powder, tuna meal and Vanderzant vitamin mix [23]. Adults were maintained with wet cottons pads soaked with a 5 % sucrose solution. For 16S rRNA gene pyrosequencing analysis, to evaluate the effect of kanamycin on mosquito microbiota, *An. gambiae* were reared in bugdorms and maintained for 10 days with 5 % sucrose solution plus kanamycin (100 μg/ml).

### *Asaia* sp. growth

*Asaia* sp. SF2.1 (GFP) [13] (hereafter, *Asaia*^gfp^) was grown 24 h at 30 °C in GLY medium (25 ml/L glycerol, 10 g/L yeast extract; pH 5.0). Cells were grown to OD^600^ = 1.0 (10^8^ recombinant bacterial cells/ml), precipitated, washed three times in 0.9 % NaCl and resuspended in 5 % sucrose solution. For monitoring long-term colonisation, the suspension was supplemented with 100 μg/ml of kanamycin to avoid plasmid loss from bacterial cells.

### Semi-field set up

The study was carried out at the confined release facility of the Department of Experimental Medicine, University of Perugia. Three large experimental cages (A-B-C) of 15.9 m^3^ each were located in a 6.68 × 3.80 × 3.00 m chamber, with complete control of environmental conditions. Briefly, a 24 h light cycle provided by four ceiling lights, dawn lasted for 30 min, full light lasted for 11.5 h and twilight lasted for 1 h and 30 min of fading ceiling light from full light to minimum power simulating sunset. Each large cage was equipped with clay resting shelters kept humid and swarming stimuli consisted of a square arena made of contrasting black and white ground marks [22, 24].

### *Asaia* sp. horizontal and vertical transmission

Horizontal spreading of *Asaia*^*gfp*^ through mosquito populations was achieved by either the release in the large cages of colonised males or by infected cotton pads. Paratransgenic *An. stephensi* and *An. gambiae* mosquitoes were obtained by oral infection of newly emerged males in bugdorms with cotton pads soaked with 5 % sucrose solution enriched with 10^8^ recombinant *Asaia*^gfp^ cells/ml and kanamycin (100 μg/ml) for 5 days. Colonised males were marked with pink fluorescent powder prior to their release in the large cages, as previously described [24]. Non-colonised males from the same batch were maintained in the same conditions. The three semi-field cages were populated with *An. stephensi* as follows: 200 newly emerged females and 200 5 day-old non-colonised males prior the release of 12 and 36 *Asaia*^gfp^-colonised males, in cage A and B, respectively. Experiments with *An. gambiae* mosquitoes were performed using the same experimental design, but only with the lowest amount of paratransgenic males (12 males in cage A). Cage C represents the negative control without insertion of paratransgenic males.

Mosquitoes were maintained with cotton pads soaked with a solution composed of 5 % sucrose, 10 % peach juice, kanamycin (100 μg/ml) and methylparaben (0.1 %) as preservative. To assess *Asaia*^gfp^ horizontal transmission, 50 females and 50 males from each cage sampled at 5, 12 and 20 days after the start of the experiment were dissected under a stereomicroscope and their guts investigated by fluorescent microscopy (Nikon Eclipse TE 2000-U); some samples were additionally analysed by PCR as described below.

To evaluate *Asaia*^gfp^ horizontal transmission in *An. gambiae* population by infected cotton pads, a cohort of newly emerged 350 males or 350 females was introduced in cage A and B, respectively. Cage C hosted 350 females and 350 males from several cohorts of individuals of different ages: in order to establish it, newly emerged adults were constantly reintroduced to maintain the density and the age distribution as stable as possible, for a month and a half before the start of the experiment. Each semi-field cage contained two uninfected cotton pads soaked with a solution composed of 5 % sucrose, 10 % peach juice, kanamycin (100 μg/ml) and methylparaben (0.1 %), and one supplemented with 10^8^*Asaia*^gfp^ cells/ml. Bacterial spread was assessed after 5 and 12 days after the release. Paternal and maternal contributions to *Asaia*^gfp^ transmission were analysed by releasing 5 day-old naïve females or males into cages A and B, respectively. Prior to the release, all the infected feeding stations were removed from cages in order to restrict co-feeding transmission routes. Mosquitoes were allowed to mate for 24 h.

To assess *Asaia*^gfp^ vertical transmission route at the end of the above described trials, females were collected and blood-fed through a Hemotek PS5 membrane feeder (Discovery Workshops, UK) at 37 °C. Engorged female mosquitoes were provided with wet filter paper for oviposition: laid eggs were floated in larval pans with breeding water containing 100 μg/ml of kanamycin, and maintained as above. Adults and 4^th^ instar larvae, collected immediately after eclosion, were sampled and screened by fluorescent microscopy and by PCR. Data were validated by statistical analysis using G test [25] and Bonferroni post-hoc test and performed in R (http://www.r-project.org/).

### DNA extraction, PCR analysis and metagenomic library preparation

Prior to DNA extraction, mosquitoes were surface-sterilised by immersion in 70 % ethanol and washed in PBS for three times, then processed with an automatic tissue homogeniser (Precellys 24, Bertin Technologies SAS, Villeurbanne, France). Genomic DNA was extracted using a JetFlex Genomic DNA Purification kit (Genomed, Lohne, Germany) according to the manufacturer’s instructions. Samples of 5 individuals were pooled together for 16S DNA pyrosequencing. Molecular analysis of *Asaia*^gfp^-infected mosquitoes was performed by PCR using the following *Asaia*^gfp^ specific oligonucleotide primers (200 nM): FOR: 5'-CAA GAG TGC CAT GCC CGA AGG-3' and REV: 5'-GAC AGG GCC ATC GCC AAT TGG-3'. PCR was performed using the DreamTaq DNA polymerase kit (Thermo Fisher Scientific, Waltham, Massachusetts, USA) according to the manufacturer’s protocol; 50 ng of genomic DNA was amplified with an initial denaturation at 94 °C for 3 min, followed by 30 cycles consisting of denaturation at 94 °C for 30 s, annealing at 60 °C for 30 s, extension at 72 °C 30 for s, and ultimately a final step at 72 °C for 10 min.

Paired-end 16S community sequencing on the Illumina MiSeq platform was performed by Polo d’Innovazione di Genomica, Genetica e Biologia, Perugia, using bacteria/archaeal degenerate primers 515 F/806R [26] to target the 16S V4 regions. 50 ng of gDNA was used for PCR amplification in 25 μl reaction volume, containing 2 μM AmpliTaq Gold 360 Master Mix (Applied Biosystem, Foster City, California, USA) and 5 μM each of the oligonucleotide primers. All amplifications were performed in a T100 Thermal Cycler (BIO-RAD, CA, USA) with an initial step at 94 °C for 3', followed by 35 cycles: 94 °C for 45 s, 55 °C for 1 min, and 72 °C for 1.5 min, and a final step at 72 °C for 10 min. Metagenomic libraries were prepared using the Nextera XT protocol (Illumina, San Diego, California, USA). Briefly, 1 ng of the purified amplicons were tagmented by the transposon, amplified via a limited-cycle PCR program that also adds index 1 (i7) and index 2 (i5), size-exclusion purified by Agencourt beads (Beckman Coulter, Brea, California, USA), normalised and pooled. The samples libraries were sequenced in a 2 × 250 PE using MiSeq Reagent Kit v2.

### Metagenomic data analysis

Metagenomic raw data were cleaned first by removing Phix contaminants using bowtie2 [27], then the sequence of the adapters and low quality scores (<20) were trimmed by Trimmomatic [28]. Cleaned reads were used to assemble the amplicons using Pear [29]. Amplicons were dereplicated, sorted and clustered to identify the OTU by vsearch (https://github.com/torognes/vsearch). OTU taxonomy was determined by a basic local alignment with BLASTn of amplicons, against the SILVA database v.119 [30]. The abundance of all OTUs identified was calculated by the alignment of the raw amplicons to the OTU references using vsearch. Normalisation and evaluation of relative abundance were performed by R (http://www.r-project.org/).

## Results and discussion

**Fig. 2:**
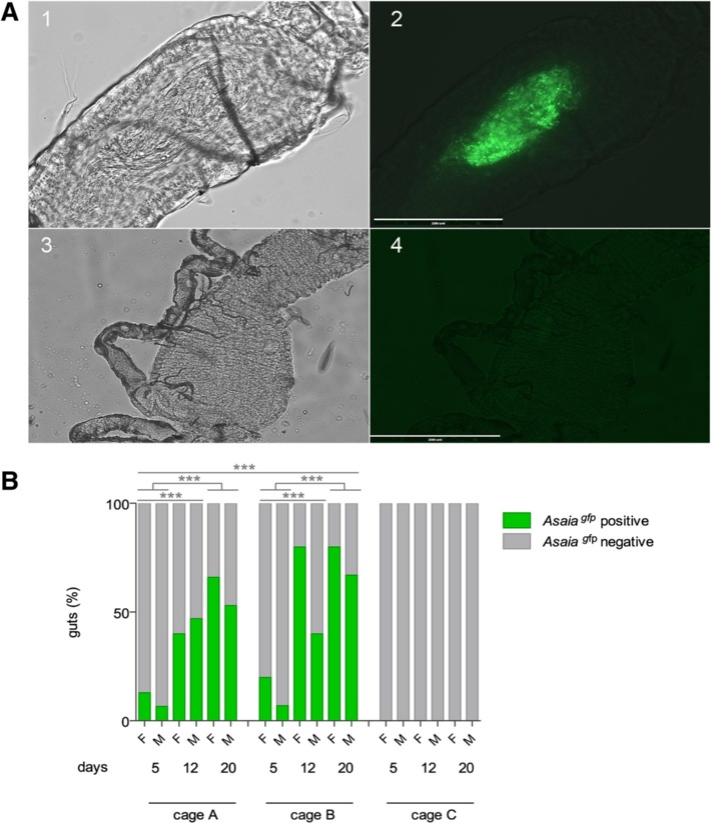
*Asaia* ^gfp^ visualisation in recipient mosquito population (**a**) and horizontal transmission analysis in large cages in *An. stephensi* (**b**)*.* **a** Optical (1, 3) and fluorescent (2, 4) images of the midgut of a female *An. stephensi. Asaia* ^gfp^ cells are clearly visible into mosquito midgut (2) from cage A after 5 days from the release of colonised males compare to mosquito from control cage C (4). Scale-bar: 200 μm. **b** Percentages of *Asaia* ^*gfp*^ positive and negative *An. stephensi* mosquitoes (female: F and male: M) in large cages at different days after the release of 12 and 36 paratrangenic males in cage A and B, respectively. Cage C represents the negative control. Means of three independent replicates are represented. Asterisks represent statistical significance (*P* < 0.001) as determined by comparisons using G-test and Bonferroni post-hoc test

**Fig. 3:**
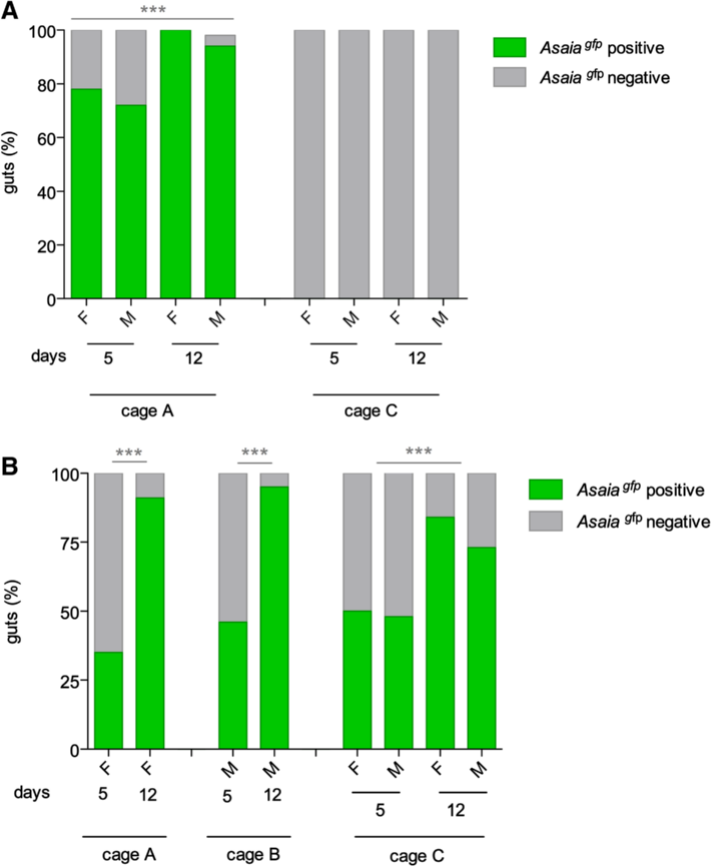
*Asaia* ^gfp^ horizontal transmission in large cages in *An. gambiae*. **a** Percentages of *Asaia* ^*gfp*^ positive and negative *An. gambiae* mosquitoes (female: F and male: M) in large cages at different days after the release of 12 paratransgenic males in cage A. Cage C represents the negative control. **b** Percentages of *Asaia* ^*gfp*^ positive and negative *An. gambiae* mosquitoes infected through cotton pads. Cages A and B host female and male recipient populations, respectively, while Cage C was populated by cohorts of mosquitoes of different ages. Mean of three independent replicates are represented. Asterisks represent statistical significance (*P* < 0.001) as determined by comparisons using G-test and Bonferroni post-hoc test

Confined semi-field conditions were used to evaluate horizontal and vertical transmission of *Asaia* expressing the green fluorescent protein (*Asaia*^gfp^) in *Anopheles* mosquitoes by the release in the large cages of paratransgenic males or infected feeding stations. *Asaia*^gfp^ spreading by paratransgenic males was evaluated in *An. stephensi* and for the first time in *An. gambiae* mosquitoes*.* The diffusion of the recombinant *Asaia* strain was evaluated by fluorescent microscopy for the presence of *Asaia*^gfp^ in the mosquito midgut (Fig. 2a). The results showed that in *An. stephensi* the percentage of mosquitoes infected with *Asaia*^gfp^ increased markedly over time and reached on average 64 % and 73 % after 20 days from the release of 12 and 36 paratransgenic males, respectively (G = 44.9, 2 d.f., *P* = 1.7e-10); Fig. 2b). The ability of *Asaia*^gfp^ to spread through the mosquito population shows a higher impact on females (G = 8.9, 1 d.f., *P* = 0.002). Similar results were obtained also in *An. gambiae* where on average 98 % of *Asaia*^gfp^ positivity was reached in just 12 days after the release of only 12 paratransgenic males (G = 113.78, 1 d.f., *P* < 2.2e-16) with no significant difference in the infection rate between male and female mosquitoes (G = 1.33, 1 d.f., *P* = 0.24) (Fig. 3a).

The trial involving horizontal bacterial transmission through sugar feeding station provided outcomes of a notable diffusion rate of *Asaia* in *An. gambiae* population and in the environment. Two uninfected and one *Asaia*^gfp^-infected feeding stations were introduced in each large cage. After two days all the feeding stations were screened for *Asaia*^gfp^ infection, resulting all positive (data not shown). Cages A, B and C were populated with only females, males or assorted stable-age mosquito population, respectively. The rate of *Asaia*^gfp^-infected mosquitoes increased over time and reached 91 %, 95 % and on average 79 % respectively, at 12 days post-release, thus indicating a successful horizontal transmission within the recipient population (G = 15.1, 1 d.f., *P* = 9.71 e-05) (Fig. 3b).

**Fig. 4:**
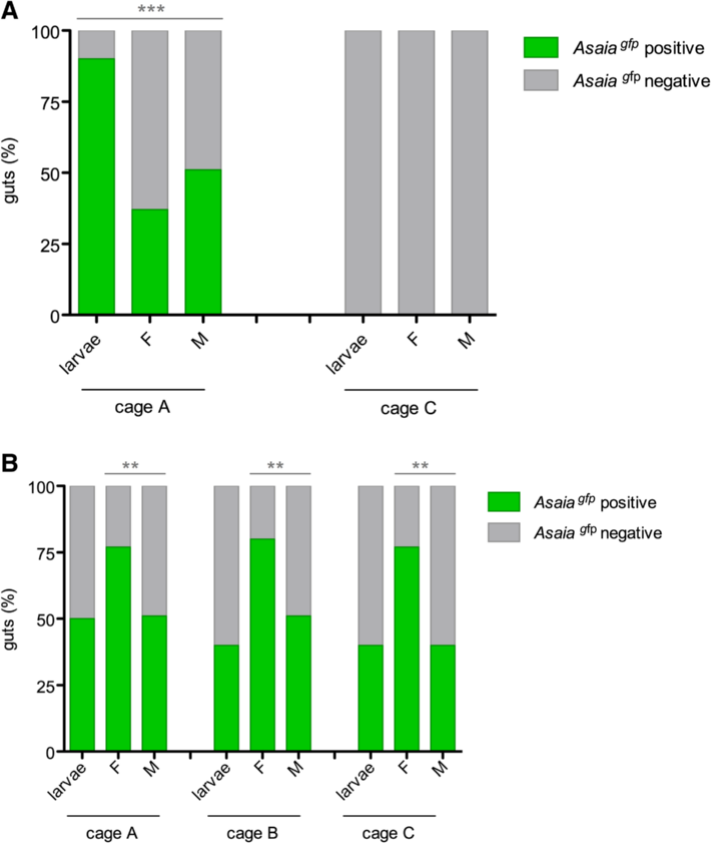
*Asaia* ^gfp^ vertical transmission in large cages in *An. gambiae*. Percentages of *Asaia* ^*gfp*^ positive and negative mosquitoes of the F1 generation of *An. gambiae* infected by paratransgenic males (**a**) and through sugar feeding station (**b**). Larvae and newly emerged female (F) and male (M) mosquitoes were analysed by fluorescent microscopy. Mean of three replicates are represented. Asterisks represent statistical significance (*P* < 0.01) as determined by comparisons using G-test and Bonferroni post-hoc test

In *An. gambiae*, the efficiency of *Asaia*^gfp^ to spread vertically and trans-stadially was additionally evaluated. Offspring of *An. gambiae* females infected in large cages by means of paratransgenic males was analysed for *Asaia*^gfp^ presence. Recombinant *Asaia* was detected in 78 % of the 4th instar larvae and in 44 % on average of the newly emerged male and female adults with respect to the control (G = 235.78, 1 d.f., *P* < 2.2e-16) (Fig. 4a). Additionally, the paternal and maternal contributions in the experimental set-up with feeding stations soaked with *Asaia*^gfp^ was investigated. In order to do this, infected mosquitoes from each cage were allowed to mate with 5 day-old uninfected mosquitoes of the opposite sex for 24 h and then to lay eggs. Fourth instar larvae were screened, reporting *Asaia*^gfp^ positivity of 50 %, 40 % and 40 % from cage A, B and C, respectively. Moreover, on average 64 %, 66 % and 59 % of adults were colonised by *Asaia*^gfp^. Differences among female and males adults were analysed, reporting a significant difference of *Asaia*^gfp^ presence (G = 8.9, 1 d.f., *P* = 0.002) (Fig. 4b).

Overall, our data are consistent with the preliminary observations obtained in previous studies performed in small cages with *An. stephensi* [18]. Additionally, this study contributes to a better understanding transmission routes and employed vectors. The strong ability of modified *Asaia* to be horizontally spread in different populations, through the release of previously infected males or through feeding stations in populations of both *An. stephensi* and *An. gambiae* is demonstrated. At the same time, our data suggest intrinsic behavioural and ecological differences between the two vector systems. The slightly lower and delayed rate of infection of *An. stephensi* compared to *An. gambiae* has been addressed to the internal arrangement of the large cages, optimised for swarming and mating behaviour of *An. gambiae*. Thus, since horizontal spreading of *Asaia*^gfp^ mainly relies on co-feeding and mating, the behaviour of the mosquitoes in this semi-field condition may have limited the second route of infection. For these reasons, the analysis of the first generation of *An. stephensi* has been prevented.

The possibility to release non-biting paratransgenic males in open field will circumvent the concerns of releasing bacteria-transmitting females, being consistent with safety requirements related to the use of paratransgenesis to reduce vector competence. Nevertheless, complexities could still arise given the fact that this practice may result in the overall increase of the mosquito population density in a given area. Therefore, we also propose an alternative approach for *Asaia*^gfp^ transmission assessment, whose introduction does not imply the release of mosquitoes, and recombinant bacteria were introduced in large cages by means of feeding stations. Both pathways definitely demonstrate the high efficiency of *Asaia* to diffuse and colonise mosquito populations in large environment with respect to small cages. This effectiveness explores both horizontal, mainly by synergistic co-feeding and mating, and vertical diffusion pathways of both paternal and maternal contribution, despite the introduction procedures applied. Our data, obtained by exploiting fluorescence marked bacteria, lay the foundation for further applications of *Asaia* as paratransgenic tool.

**Fig. 5:**
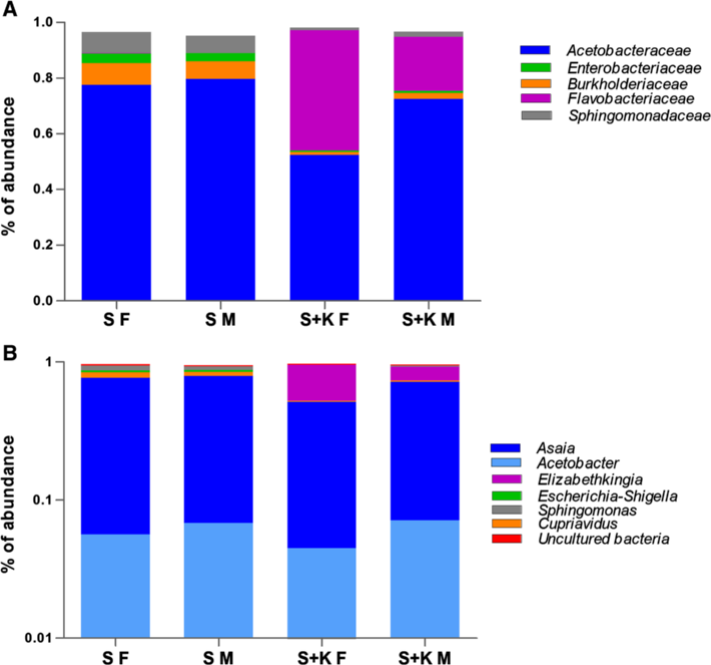
Interference evaluation of antibiotic selection on *Asaia* ^gfp^ transmission. Proportions of OTU abundance are represented at the family (**a**) and genus (**b**) taxonomic level in males (M) and females (F) of *An. gambiae*. Families and genera with abundance of >1 % in at least one sample are presented. *Abbreviations*: S, mosquitoes fed with sugar meal; S + K, mosquitoes fed with sugar meal supplemented with kanamycin

Finally, we have also addressed the question related to the use of antibiotic in the feeding stations for the maintenance of recombinant bacteria. To determine whether or not the use of kanamycin in feeding stations affects the ability of *Asaia*^gfp^ to colonise mosquito populations by altering the native microbiota, we performed a 16S rRNA gene pyrosequencing of mosquitoes kept under antibiotic selection. The metagenomic analysis clearly showed that kanamycin partially affects mosquito microbiota at both family and genus taxonomic levels (Fig. 5, Additional file 1: Table S1 and Additional file 2: Figure S1). Nevertheless, out of several tens of bacteria genera investigated, only species of the family Flavobacteriaceae and, specifically of the genus *Elizabethkingia*, seem to have a notable bloom mainly in female mosquitoes kept under antibiotic selection (Fig. 5a, b and Additional file 1: Table S1). This is in agreement with a previous report describing *Elizabethkinghia* sp. as a dominant bacteria species present in the gut of the malaria vector *An. gambiae* with a broad antibiotic resistance [31]. Our data, although showing that the mosquito microbiota is partially affected by the antibiotic selection, clearly indicate that kanamycin treatment does not account for the success of *Asaia* transmission, whose persistence is constant and clearly demonstrated.

## Conclusions

The ability of paratransgenic approaches to control malaria and other mosquito-borne diseases is very promising, disclosing a concrete applicative prospect. We report here results from the first known paratransgenic trial performed in large cages aimed at testing the feasibility of this approach. The success of paratransgenesis obviously depends on a variety of factors. Nevertheless, our findings support the applied perspective involving the use of *Asaia* as a promising tool and further demonstrate the great utility of confined environments to define the most efficient methodologies for an in-depth evaluation of technologies transition from laboratory to field employment. The field release of paratransgenic mosquitoes imposes a rigorous risk assessment framework coherent with a strict regulatory system, appropriate to national and international guidelines. Evaluation of the risks and benefits of this strategy is required. Investigation of hazards and safety related concerns [32], together with implementation of authorised ongoing projects (transgenic and/or *Wolbachia*-transinfected mosquitoes [33]) will lay the basis for a solid regulatory oversight of the paratransgenic program, and ultimately, to allow its field trials. Since *Asaia* has been recently detected in several insect vectors [34–37], these data provide crucial clues applicable toward multiple paratransgenic targets in the control of a wide spectrum of vector borne diseases.

## Additional files

Additional file 1: Table S1. Bacterial composition (number of reads) at phylum, class, order, family, genus level for sugar -fed and antibiotic-treated *An. gambiae* mosquitoes. (XLS 106 kb) Additional file 2: Figure S1. Interference evaluation of antibiotic selection on *Asaia* ^gfp^ transmission. (DOCX 214 kb)
