# Supplementary material for: Paratransgenesis to control malaria vectors: a semi-field pilot study
Source: Parasit Vectors. 2016 Mar 10;9:140. doi: 10.1186/s13071-016-1427-3 (PMC4787196; doi:10.1186/s13071-016-1427-3)
Supplement: Additional file 2: Figure S1. — Interference evaluation of antibiotic selection on Asaia gfp transmission. (DOCX 214 kb) [file 13071_2016_1427_MOESM2_ESM.docx]

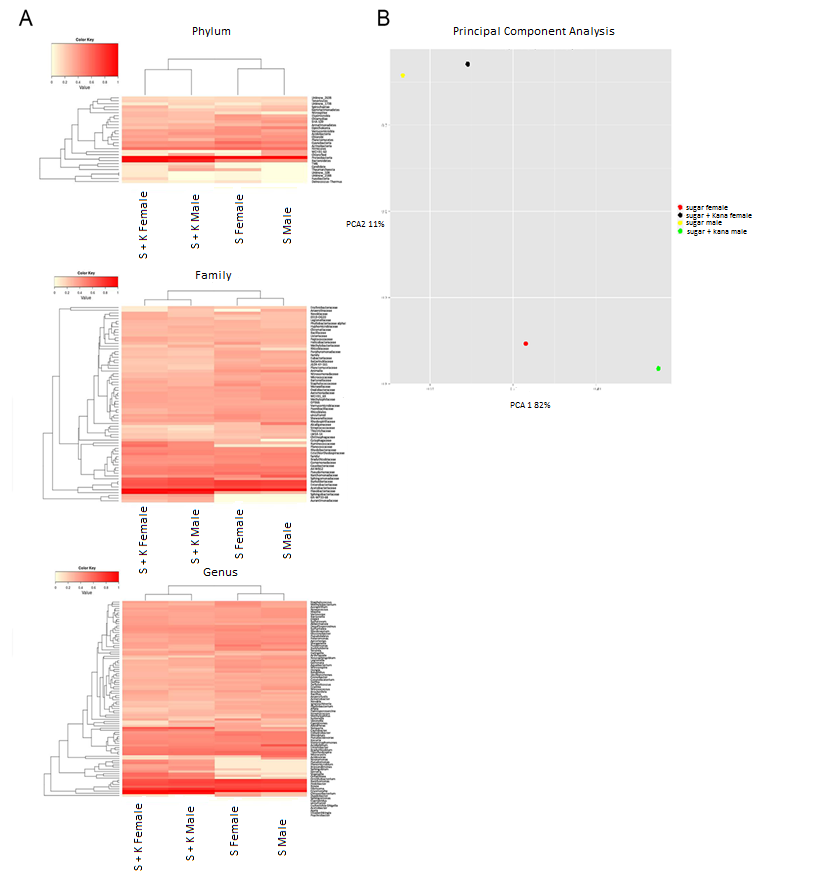


**Additional File 2: Figure S1**

**Interference evaluation of antibiotic selection on *Asaia ^gfp^* transmission.** Panel A describes the relative abundance of bacterial genera in heatmaps, representing the proportions of OTUs at the phylum (upper), family (middle) and genus (bottom) levels. Panel B shows the Principal Component Analysis (PCA) plots of gut bacteria diversity (colored dots represent mosquito samples).
